# Magnetic signature reproduction of ferromagnetic ships at arbitrary geographical position, direction and depth using a multi-dipole model

**DOI:** 10.1038/s41598-023-41702-4

**Published:** 2023-09-05

**Authors:** Miroslaw Woloszyn, Jarosław Tarnawski

**Affiliations:** Gdańsk University of Technology, Gdańsk, Poland

**Keywords:** Engineering, Electrical and electronic engineering

## Abstract

The reproduction of magnetic signatures is an important issue concerning the safety of ship traffic, as well as the identification and classification of vessels. Moreover, military applications of magnetic signatures and their reproduction refer to the activation or protection against activation of magnetic naval mines. Previous works on this subject focused on recording and replicating the signatures under the same conditions as those under which they were measured, e.g., on the same ship courses. In this article, much greater capabilities of the multi-dipole model are presented, including simultaneous identification of permanent and induced magnetism. Determining the dipole values using the data from cardinal directions gives the possibility of determining the magnetic field density at any trajectory (position), direction, or depth, with further reconstruction of the entire magnetic field on the basis of residual measurements. For the purpose of this article, a numerical test model of a corvette-type ship has been modelled in Opera simulation software for different geographical positions. The synthetic data from the simulator served as the data source for determining the parameters of the multi-dipole model and the reference data for the verification of the signatures reconstructed for other positions, directions, and depths than those used to determine the model parameters. To determine all permanent magnetization components, data sets were used for two different values of the external magnetic field vertical component. Finally, as a culmination of the demonstration of model universality, the entire magnetic field around the ship was reproduced for different control points on Earth, and for different courses and depths. Investigating the possibility of reconstructing the magnetic signature at a different geographic location than the place where the measurement was made for model synthesis is the main original issue considered in this paper.

## Introduction

A ship constructed of ferromagnetic steel disturbs the Earth’s magnetic field. This disturbance is called the ship’s magnetic signature and due to it the ship can be destroyed by marine mines^1,2^. The authors of^3^ presented the system which uses a bottom looking sonar, a Real-time Tracking Gradiometer (RTG), and an Electro-Optic Imager (EOI). The above problem is serious and dangerous for all ferromagnetic ships. The main sources of the ship’s magnetic signature were described in^4,5^. There are several analytical models of the magnetic field generated by a sphere^6,7^ and a prolate ellipsoid^8^. The spheroidal harmonic expansion coefficients of the magnetic scalar potential have been applied in the mathematical model of a prolate spheroidal marine vessel^9–11^. The authors of these articles discussed several strategies to improve the estimation of model parameters based on the measured field data. They achieved a series expansion which describes the ship’s signature in its near field very accurately. When sufficient information is provided by near field measurements, then the far field can also be accurately represented. The disadvantage of that model is that it reproduces the signature only along the lines for which the measurements have been carried out. Modelling of the magnetic field of a given ship is complicated due to its shape and magnetic properties of ship’s steel. The ship constructed of ferromagnetic steel has two types of magnetization: induced^12^ and permanent. The ship's magnetic field can be modelled using a set of induced and permanent dipoles and then calculated using the FEM method^13–15^. In^16^, the ship’s multi-dipole model was compared with the physical model in North–South and South–North direction with good results. In^17^, the authors presented a multi-dipole magnetic model of the ship using the induced and permanent magnetic dipoles, and compared it with a real warship with good results. The authors of^18,19^ presented a multi-dipole model which allows to reproduce the ship’s magnetic signature for any direction and depth. That model was also compared with success with the real marine ship *Zodiak*^20^. The magnetic signature can be decomposed into parts referring to induced and permanent magnetization^21,22^. In^21^, the authors introduced an algorithm for this signature decomposition. The applied method was based on measuring the magnetic field in four cardinal magnetic directions of the ship: 0°, 90°, 180°, 270°. The permanent magnetization can be assumed independent on the external field, while the induced magnetization depends on the strength and direction of the Earth’s magnetic field. After introducing the effective permanent dipole moment by adding the induced vertical dipole moment to the permanent vertical dipole moment, the authors of^21^ solved an inverse problem and presented the ship’s magnetic signature for another direction in the same area.

Important properties of the multi-dipole magnetic model which were recognized during the previous research performed by the authors of this article are:

- the magnetic signatures can be reconstructed for an arbitrary direction of the ship,
- the magnetic signatures can be reconstructed for an arbitrary water depth, but greater than the measuring depth

The ability to determine the level of magnetic field density at different depths and for different directions is a very important feature of the multi-dipole model. This property is also used to validate the model. When the values determined from the model (with parameters from a different geographic location and depth) coincide with the values determined from the reference source (measurement or FEM), the validity of the model can be confirmed.

This virtual magnetic model reconstructs the ship’s magnetic field very well not only along specific lines but also at any point of the surface (*z*- constant). In this article, the authors extend the analysis of the functionality of the multi-dipole model to include the potentially extremely useful feature of transferring a magnetic signature acquired in one geographic location to another. The analysis undertaken in this article is an original contribution to establishing the conditions under which signature transformation is possible. On the basis of the magnetic fields generated by the numerical model of a ship built in Opera 3D for six different points on Earth’s surface (2 Poles, Equator, and 3 points located in the northern and southern hemispheres), the authors investigate the possibility to reconstruct the magnetic signatures. The simulations of ship’s magnetic fields were carried out in Simulia Opera 3D^23^ using the thin plate boundary condition^14,24^. Material isotropy of the ship was assumed in the numerical simulations. The knowledge about the ship’s magnetic signature at an arbitrary point can be used to design a degaussing system which would minimize the magnetic signature^25,26,30^.

## Multi-dipole ship model

**Figure 1:**
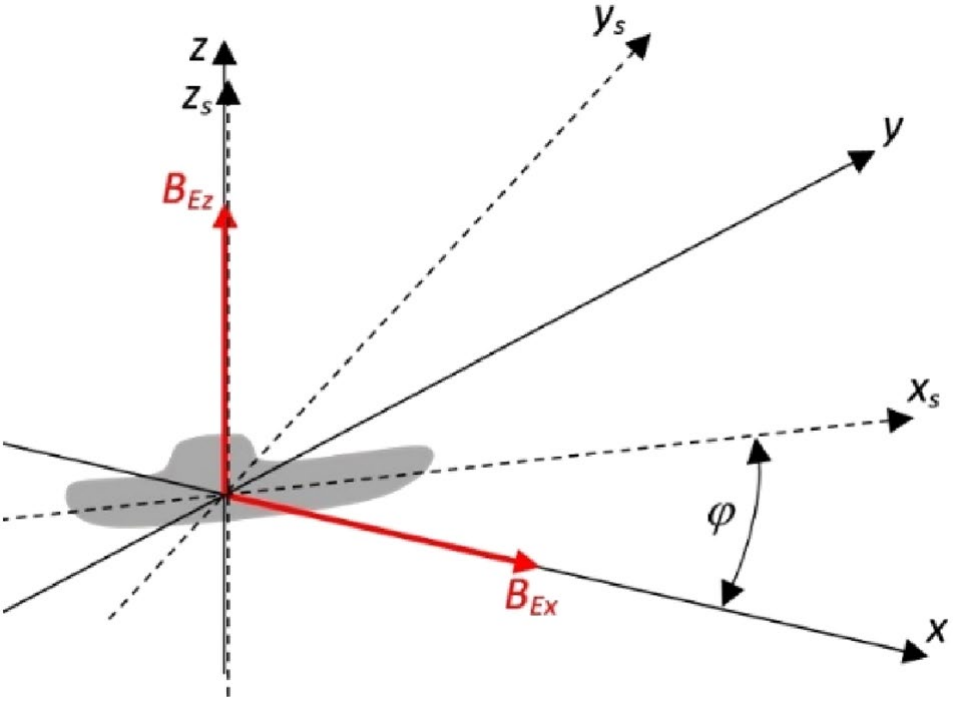
The Cartesian coordinate system (*x*, *y*, *z*) and the ship’s coordinate system (*x*_*s*_, *y*_*s*_, *z*_*s*_).

The multi-dipole model is capable of reconstructing the induced and permanent form of magnetism. The term induced dipoles refers to the dipoles used to reconstruct induced magnetism, and the term permanent dipoles refers to the dipoles used to describe permanent magnetism. In the multi-dipole model, the parameters of the induced and permanent dipoles are determined on the basis of the magnetic signatures of the ship along three lines under the ship in four magnetic directions. Based on the signals in four directions along three lines (Port, Keel, Starboard), the magnetic moments of the induced and permanent dipoles were calculated for the ship located in the region with the Earth's magnetic flux density vector components (*B*_*Ex*_, 0, *B*_*Ez*_) (Fig. 1). The magnetic flux density vector generated at point (*x, y, z*) by the *n*-th dipole with coordinates (${x}_{n}$, ${y}_{n}$, ${z}_{n}$) is^5^:

1 $${\mathbf{B}}_{d,n}=\frac{{\mu }_{0}}{4\pi {R}_{n}^{3}}\left({\mathbf{R}}_{n}^{T}{\mathbf{M}}_{n}{\mathbf{R}}_{i}\frac{3}{{R}_{n}^{2}}-{\mathbf{M}}_{n}\right)$$

where

2 $${\mathbf{M}}_{n}=\left[\begin{array}{c}{m}_{x,n}\\ {m}_{y,n}\\ {m}_{z,n}\end{array}\right]$$

Here, ${\mathbf{M}}_{n}$ is the vector of magnetic dipole moment of the *n*-th dipole, and ${\mathbf{R}}_{n}$ is the distance vector of the point (*x, y, z*) from the *n*-th dipole position with coordinates (${x}_{n}$, ${y}_{n}$, ${z}_{n}$).

3 $${\mathbf{R}}_{n}=\left[\begin{array}{c}(x-{x}_{n})\\ (y-{y}_{n})\\ (z-{z}_{n})\end{array}\right]$$

The total vector of the magnetic field density generated by all dipoles is:

4 $${\mathbf{B}}_{{\varvec{d}}}\left(x,y,z\right)=\sum_{n=1}^{N}{\mathbf{B}}_{d,n}$$

where *N* is the total number of permanent and induced dipoles.

The multi-dipole model distinguishes between permanent and induced dipoles. The parameters of all dipoles are calculated for ship’s course *φ* = 0°. The positions of the dipoles are fixed, but they have to be transformed into the Cartesian coordinate system, along with the components of magnetic moments of permanent and induced dipoles regarding the ship’s course *φ*^18^. The components of the permanent magnetic dipole moment and the induced magnetic moment in the Cartesian coordinate system are given by formula (5) and formula (6), respectively^19^.

5 $${\mathbf{M}}_{P,i}=\left[\begin{array}{*{20}l}{m}_{xP,i}cos\varphi -{m}_{yP,n}sin\varphi \\ {m}_{xP,i}sin\varphi +{m}_{yP,n}cos\varphi \\ {{m}_{zP,i}1}_{z}\end{array}\right]$$

6 $${\mathbf{M}}_{I,j}=\left[\begin{array}{*{20}l}{m}_{I1,j}+{m}_{I2,j}{cos}^{2}\varphi \\ {m}_{I2,j}sin\varphi cos\varphi \\ {{m}_{I3,j}1}_{z}\end{array}\right]$$

where ${m}_{xP,i}$, ${m}_{yP,i}$, ${m}_{xP,i}$ are the components of the permanent magnetic moment vector of the *i*-th dipole, $\varphi$ is the ship’s course (Fig. 1), *m*_*I*1*,j*_, *m*_*I*2*,j*_, and *m*_*I*3*,j*_ are the aggregated components of the induced magnetic dipole moments of the *j*-th dipole. The size of matrix **M**_*P,i*_ is 3 × *Np,* where *Np* is the number of permanent dipoles; the size of matrix **M**_*I,j*_ is 3 × *Ni,* where *Ni* is the number of induced dipoles; and the size of matrix **M**_*n*_ is 3 × (*Np* + *Ni*)*, i ∈*  < 1, *Np* > , *j ∈*  < 1, *Ni* > , *n ∈*  < 1, *Np* + *Ni* > , where *Np* is the number of dipoles.

The total magnetic moment vector is:

7 $$\mathbf{M}={\mathbf{M}}_{P}\cup {\mathbf{M}}_{I}$$

The value of the Earth’s magnetic field is weak (of several dozen μT) and therefore changes of the magnetic field inside the ship steel remain on the linear part of the magnetization characteristic. The components of the induced magnetic moment of the dipole depend proportionally on the Earth’s magnetic field (*B*_*Ex*_, 0, *B*_*Ez*_) (Fig. 1). Thanks to the linear property of this phenomenon, the values of the induced magnetic moment components of each dipole for other values of the Earth’s magnetic field (*B’*_*Ex*_, 0, *B’*_*Ez*_) can be calculated as:

8 $${m}_{I1,j}^{\prime}={m}_{I1,j}\frac{{B}_{Ex}^{\prime}}{{B}_{Ex}}$$

9 $${m}_{I2,j}^{\prime}={m}_{I2,j}\frac{{B}_{Ex}^{\prime}}{{B}_{Ex}}$$

10 $${m}_{I3,j}^{\prime}={m}_{I3,j}\frac{{B}_{Ez}^{\prime}}{{B}_{Ez}}$$

Equations (8–10) allow to scale the magnetic moments of all induced dipoles for a new value of the Earth magnetic field **B**_*E*_^′^. The permanent magnetic moments of the dipoles do not depend on the external field and direction. That makes it possible to reconstruct the ship’s magnetic signature at any point on Earth by scaling only the induced dipoles.

## The ship model and its multi-dipole equivalent

**Figure 2:**
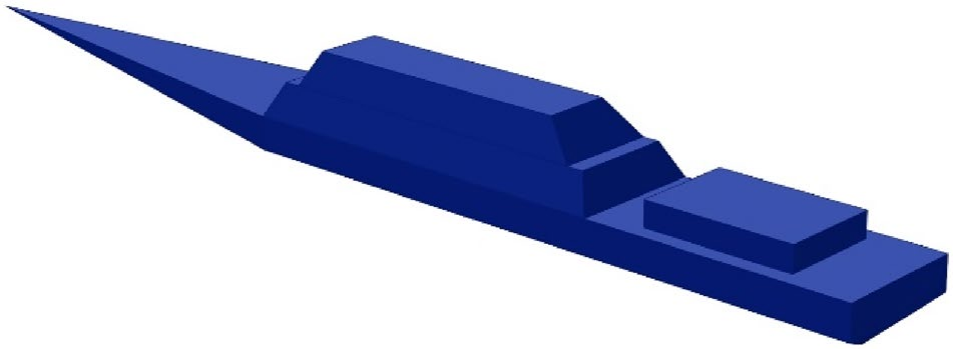
The ship model.

**Figure 3:**
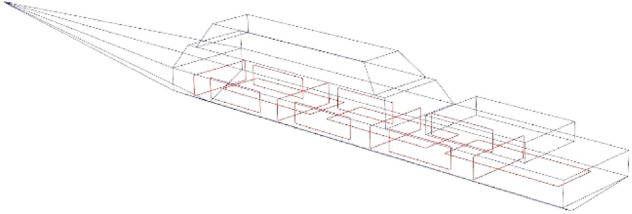
Coils inside the ship model.

The numerical model of the ship was built in the Opera 3D program. This program, using the FEM method, has a very useful property for modelling of objects with relatively small thickness of ferromagnetic material. The thin plate boundary condition implemented in this program allows FEM-based modeling of ships with good shapes^14,24^. The degree of magnetization of the ferromagnetic plates of the ship’s hull is unknown. The phenomenon of permanent magnetization of the ship’s steel is complex and the magnetization itself is constantly and slowly changing due to mechanical impacts of the ship against the water surface or against the quay. In this paper, coils inside the ship are used to simulate the permanent magnetization of the steel. A series of coils as shown in Fig. 3 were used to generate a constant magnetic field in three axes x, y, z with such values that the signature of the ship with and without permanent magnetization is clearly different. The ship’s model presented in Fig. 2 has the following dimensions: length 70 m, width 8 m, and height 9 m. The relative magnetic permeability *μ*_*r*_ = 200 and thickness 1 cm of the ferromagnetic steel were assumed. This value is one of the typical values of ship steels. However, the effect of the relative magnetic permeability of steel on the magnetic signature is significant. The great advantage of the multi-dipole model is the selection of model parameters based only on synthetic or real magnetic data without the required ship shape and relative magnetic permeability values. For the purpose of verifying the proposed method of reconstructing the ship's magnetic signatures, an equal value of relative magnetic permeability of steel was assumed in the simulation.

The magnetic fields of the ship’s model at different points on Earth were calculated by introducing appropriate values of the external field. After subtracting the total magnetic field from the Earth's field, the ship’s magnetic signature at any point on Earth was obtained. The values of FEM parameters are given in Table 1.

**Table 1:** FEM parameters for ship model.

Parameter	Value
Number of active elements	4,319,341
Number of nodes	906,792
Number of equations	766,767
Number of non-zeros	6,298,862

**Figure 4:**
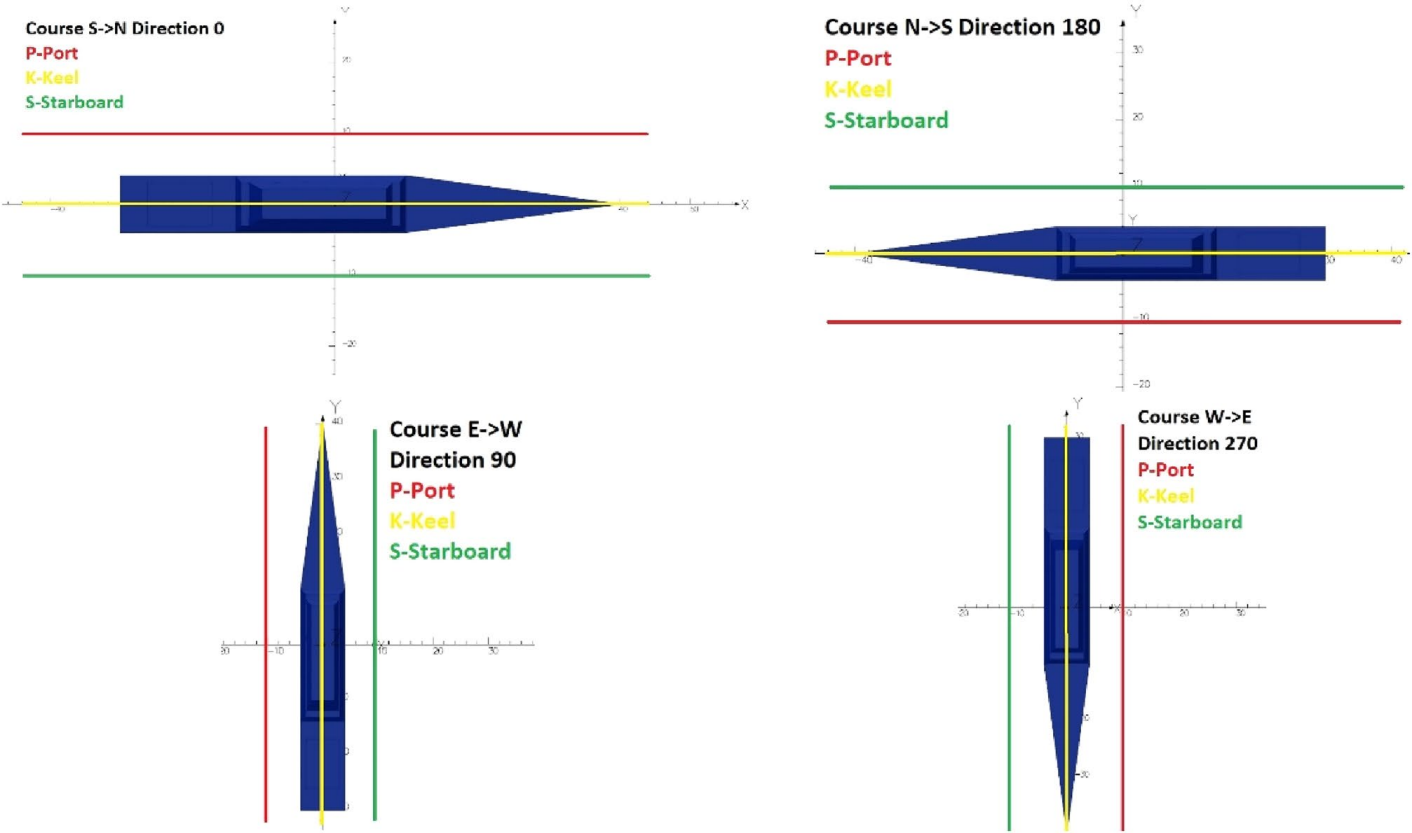
Intersection of the MFD field to obtain data along PKS tracks for different ship directions.

The data for a single scenario calculation refers to four cardinal directions (0°, 90°, 180°, 360°) and fields with dimensions of 601 × 601 points (x = − 300 m: 300 m; y = − 300 m: 300 m, every single meter) for all MFD components *B*_*x*_*, B*_*y*_*, B*_*z*_. The largest MFD values are present under the keel, but in order to correctly reconstruct the signature for a ship with both induced and permanent magnetism additionally, field values at the sides of the ship are usually measured as well. Such diverse origins of the data describing the field make it possible to obtain a robust model. The fields are crossed at PKS (Port, Keel, Starboard) lines—see Fig. 4—to establish the path data as it is on the measurement range. Thus, 601 points × 3 paths × 4 directions × 3 fields are used in one scenario, making a total of 21,636 input points. A complete input data set is made available by the authors in the form of an archive published on the^27^.

The procedure for determining the multi-dipole model parameters has been described in detail by the authors in^18^ and^19^. For the convenience of the reader, the optimization criterion is presented here as (11).

The optimization problem for a model structure described in Chapter 2 with *m* permanent dipoles and *n* induced dipoles is defined as follows:

11 $$\mathop {\min }\limits_{{\Omega \in \left\{ {\Omega_{1} , \ldots ,\Omega_{n + m} } \right\}}} {\text{J = }}\sum\limits_{comp} {\sum\limits_{pks} {\sum\limits_{dir} {\sum\limits_{j = - 300}^{300} {\left( {B_{comp,dir,pks}^{ref} (j) - B_{comp,dir,pks}^{model} (j,\Omega )} \right)^{2} } } } }$$

_subject to:_

$$\mathop \forall \limits_{{i \in (1,N_{p} + Ni)}} \Omega_{i}^{\min } \le \Omega_{i} \le \Omega_{i}^{\max } ,$$

$\Omega_{i}^{\min } ,\Omega_{i}^{\max }$ are the vectors of minimal and maximal constraint values for the decision variables related to the i-th considered dipole.

where:

$$\mathop \forall \limits_{i \in (1,m + n)} \Omega_{i} \in \left\{ {m_{x,i} ,m_{y,i} ,m_{z,i} ,x_{i} ,y_{i} ,z_{i} } \right\},comp \in \left\{ {B_{x} ,B_{y} ,B_{z} } \right\},pks \in \left\{ {P,K,S} \right\},dir \in \left\{ {0^{ \circ } ,90^{ \circ } ,180^{ \circ } ,270^{ \circ } } \right\}$$

The objective function J (11) defines the difference in matching the reference and model data in all considered directions, for paths P, K, S (9), over the length of 600 m, with the resolution of one meter for magnetic field components *B*_*x*_*, B*_*y*_*, B*_*z*_ (8). Inside the criterion function, there is the sum of squares of model and source data differences for individual magnetic field components.

Proper determination of the parameters of the multi-dipole model requires acquiring data in the four magnetic directions 0°, 90°, 180° and 270°. For the ship's 0° and 180° courses, it is possible to obtain information on the ship's longitudinal magnetization, and for the 90° and 270° directions on transverse magnetization (30). Determining the dipole model parameters associated with vertical magnetization is more complex, as it requires obtaining the ship's magnetic data for two different vertical components of the external magnetic field (30). If the ship had only induced magnetization, then data obtained only under the keel would suffice. However, in practice, every ship has a permanent magnetization, so the minimum data needed to reconstruct the signature requires acquiring data under the keel and from the starboard and port sides.

Gradient and non-gradient methods can be used to solve this nonlinear optimization task. However, the latter require significantly longer computation time, hence the gradient approach is used in practice. The Trust Region Reflective^28^ method, available in computing packages, has been tested for this purpose in many applications.

**Figure 5:**
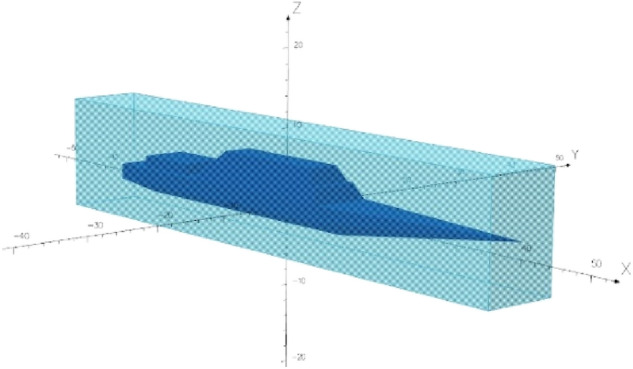
The area around the ship with possible dipole locations determined by constraints for the optimization procedure.

The solution to the optimization task has a form of six values for each of the induced and permanent dipoles. The first three parameters refer to the magnetic moment components, while the next three are the locations of the dipoles in the *x*, *y*, *z* space of the multi-dipole model. The space for the dipoles is assumed to be a cuboid described on the ship in the space limited by the ship's body as shown in Fig. 5. The dimensions of this cuboid are the constraints on the optimization procedure. The number of the dipoles depends on the measurement depth, and on the shape and dimensions of the ship. The search for correct parameterization of the model is still an open analytical task, which can be solved using regularization. Based on previous experience gained by the authors, 30 induced and 30 permanent dipoles were assumed for the model presented in this article and the measurement depth of 20 m. The results of the optimization procedure in the form of a list of parameterized dipoles can be found in the archive accompanying this publication^27^.

## Methodology of validation of ship’s magnetic reconstruction

The correctness of the new approach to the reconstruction of ship’s magnetic signatures at any point on Earth was verified for cases without and with permanent ship’s magnetization. Three simulation scenarios S1, S2, and S3 were investigated, as shown in Table 2. Scenario S1 deals only with the induced magnetism, while Scenario S2 with both induced and permanent magnetism, but using only one set of data. Finally, Scenario S3 involves the use of two data sets for vertical component determination.

**Table 2:** Calculation scenarios.

#	Type of magnetism and its components	Data source for dipoles	Data Set for calculation (files available in^27^)
S1	Induced	REF	DataS1_V1.mat DataS1_V2.mat DataS1_V3.mat DataS1_V4.mat DataS1_V5.mat DataS1_V6.mat
S2	Induced, permanent	REF	DataS2_V1.mat DataS2_V2.mat DataS2_V3.mat DataS2_V4.mat DataS2_V5.mat DataS2_V6.mat
S3	Induced, permanent	REF with REF *B*_Ez_ + 20μ_0_ μT	DataS2_V1.mat with DataS3_V1.mat DataS2_V2.mat with DataS3_V2.mat DataS2_V3.mat with DataS3_V3.mat DataS2_V4.mat with DataS3_V4.mat DataS2_V5.mat with DataS3_V5.mat DataS2_V6.mat with DataS3_V6.mat

**Figure 6:**
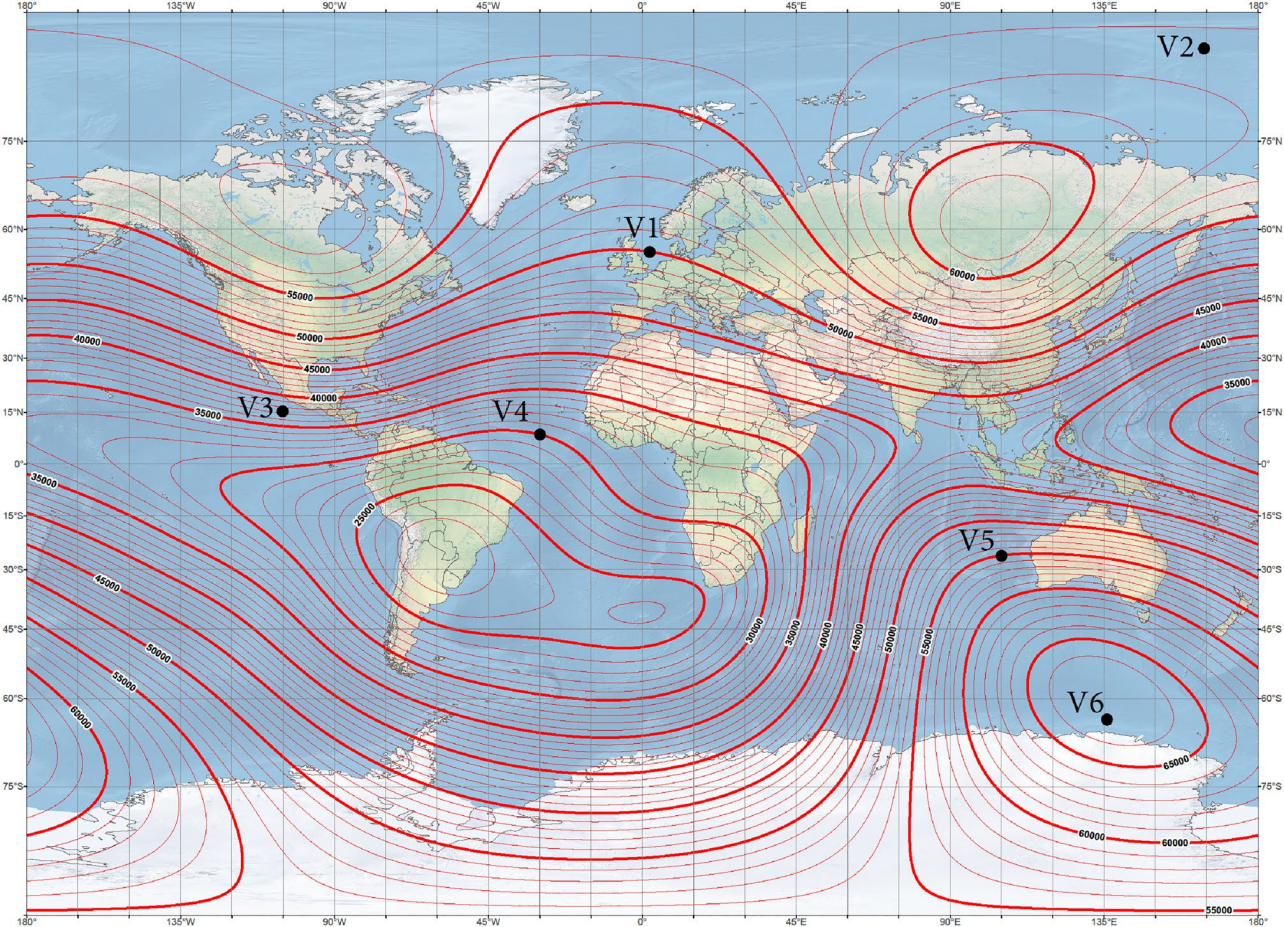
Magnetic isoclines at points V1–V6 (source: National Centers for Environmental Information^29^).

**Figure 7:**
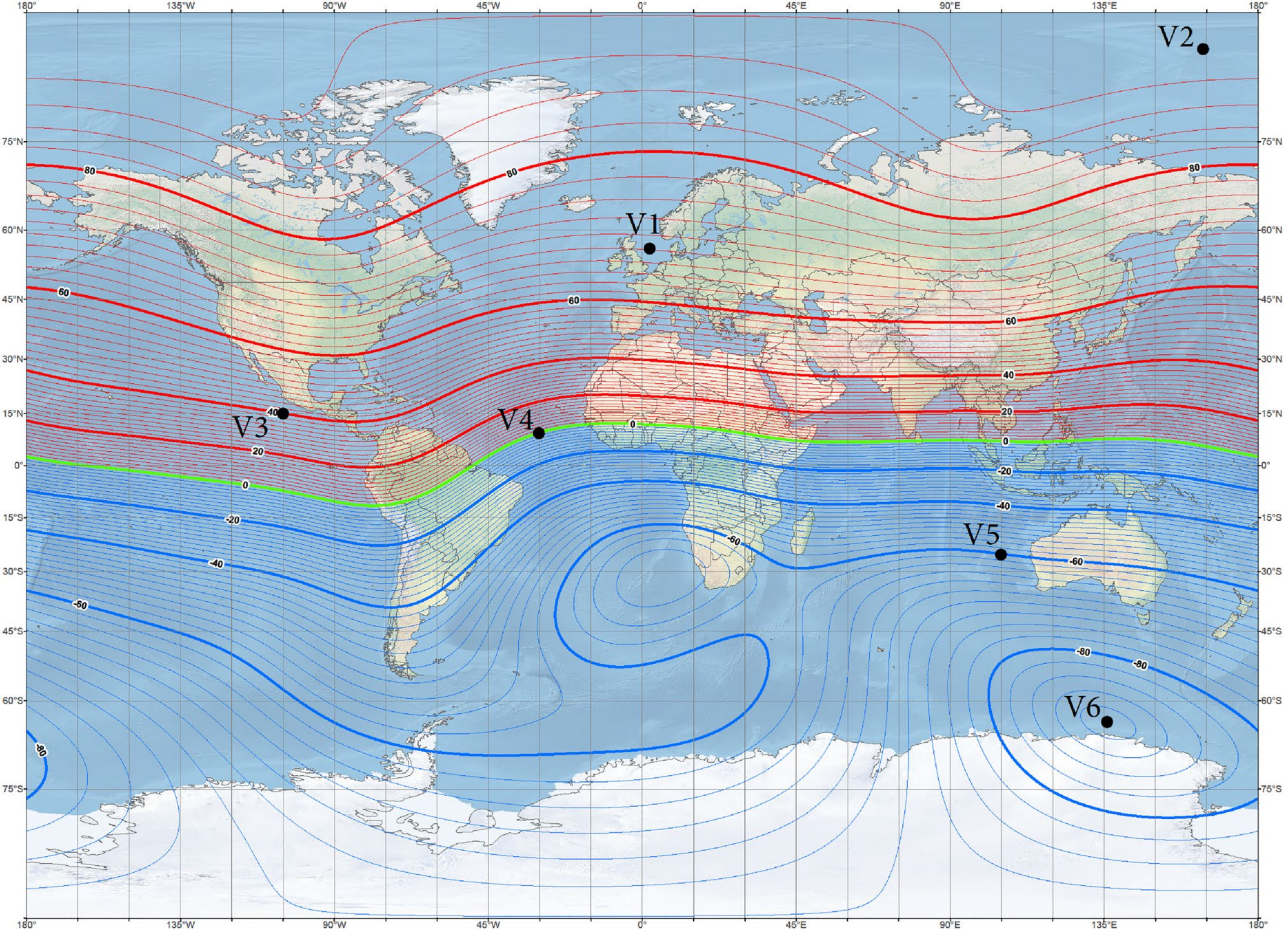
Inclination isoclines at points V1-V6 (source: National Centers for Environmental Information^29^).

In the applied methodology, on the basis of the ship’s magnetic signatures in four magnetic directions at a chosen geographical point, the parameters of the ship’s multi-dipole model (magnetic moments and positions of dipoles) were first achieved using the optimization method^28^. Next, all induced magnetic moments of the dipoles were scaled according to formulas (7–9) for each new value of the Earth magnetic field vector (new geographical point). The reconstructed ship’s magnetic signatures were validated with the magnetic signatures calculated in Opera 3D. The six points V1 ÷ V6 presented in Table 3 and on the world maps of total magnetic flux density (Fig. 6) and inclination isoclines (Fig. 7) of the Earth were adopted for the analysis. Each point was assumed as the reference magnetic field source for the calculation of multi-dipole parameters. When the multi-dipole model was obtained, the validation of magnetic signatures at all points was carried out. The validation of the reconstructed ship’s magnetic signatures without permanent magnetization is discussed in Chapter 4.1, while with permanent magnetization in Chapters 4.2 and 4.3.

**Table 3:** Geographic position and magnetic field density values at reference points.

Point	Geographic position Latitude [°], Longitude [°]	Total ambient field module [µT]	Inclination [°]	*B*_*Ex*_ [μT]	*B*_*Ey*_ [μT]	*B*_*Ez*_ [μT]	Direction [°]	Depth [m]
V1	55 N, 2 E	50	70	17.101	0.000	− 46.985	Cardinal	− 20
V2	90 N, 165 E	52.5	90	0.000	0.000	− 52.500	Cardinal	− 20
V3	15 N, 100 W	37	40	28.343	0.000	− 23.783	Cardinal	− 20
V4	10 N, 30 W	30	0	30.000	0.000	0.000	Cardinal	− 20
V5	25 S, 100 E	55	− 60	27.500	0.000	47.631	Cardinal	− 20
V6	63 S, 135 E	67	− 90	0.000	0.000	67.000	Cardinal	− 20

The results of particular simulation scenarios can be compared qualitatively in the form of graph observations, and quantitatively using the RMSE (root mean square error) and MaxAbsError indicator values on the paths. The indicator RMSE provides the information about the average result, while MaxAbsError represents the maximum error along the considered path. Taken together, these indices make it possible to evaluate the signature reconstruction.

The root mean square error is given by:

12 $$RMSE = \sqrt {\frac{1}{N}\sum\limits_{i = 1}^{N} {(ref_{i} - model_{i} )^{2} } }$$

and the MaxAbsError is given by:

13 $$MaxAbsError=\mathrm{max}\left(abs\left({ref}_{i}-{model}_{i}\right)\right), i=1..N$$

where *model*_*i*_ is the vector of *N* signature values at i-th position coordinate counted by the model, and *ref*_*i*_ is the vector of *N* reference signature values at the same position.

### Methodology of validation of ship’s magnetic reconstruction without permanent magnetization

**Figure 8:**
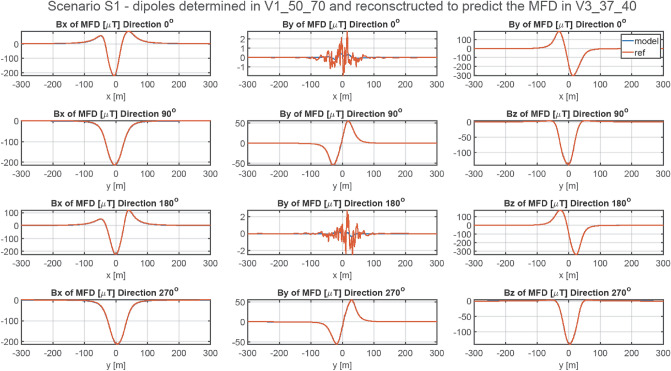
Validation of magnetic signatures at point V3 based on scaled multi-dipole parameters obtained for point V1.

**Figure 9:**
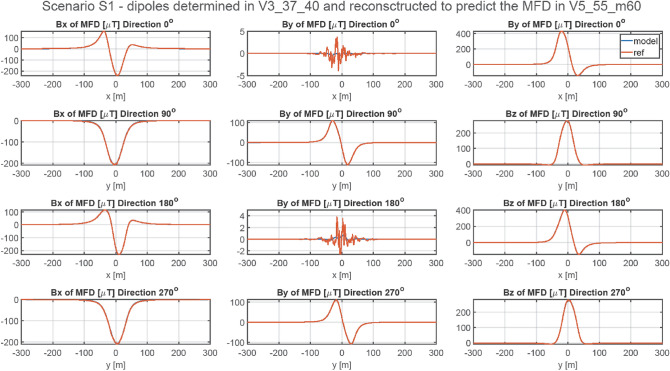
Validation of magnetic signatures at point V5 based on scaled multi-dipole parameters obtained for point V3.

**Figure 10:**
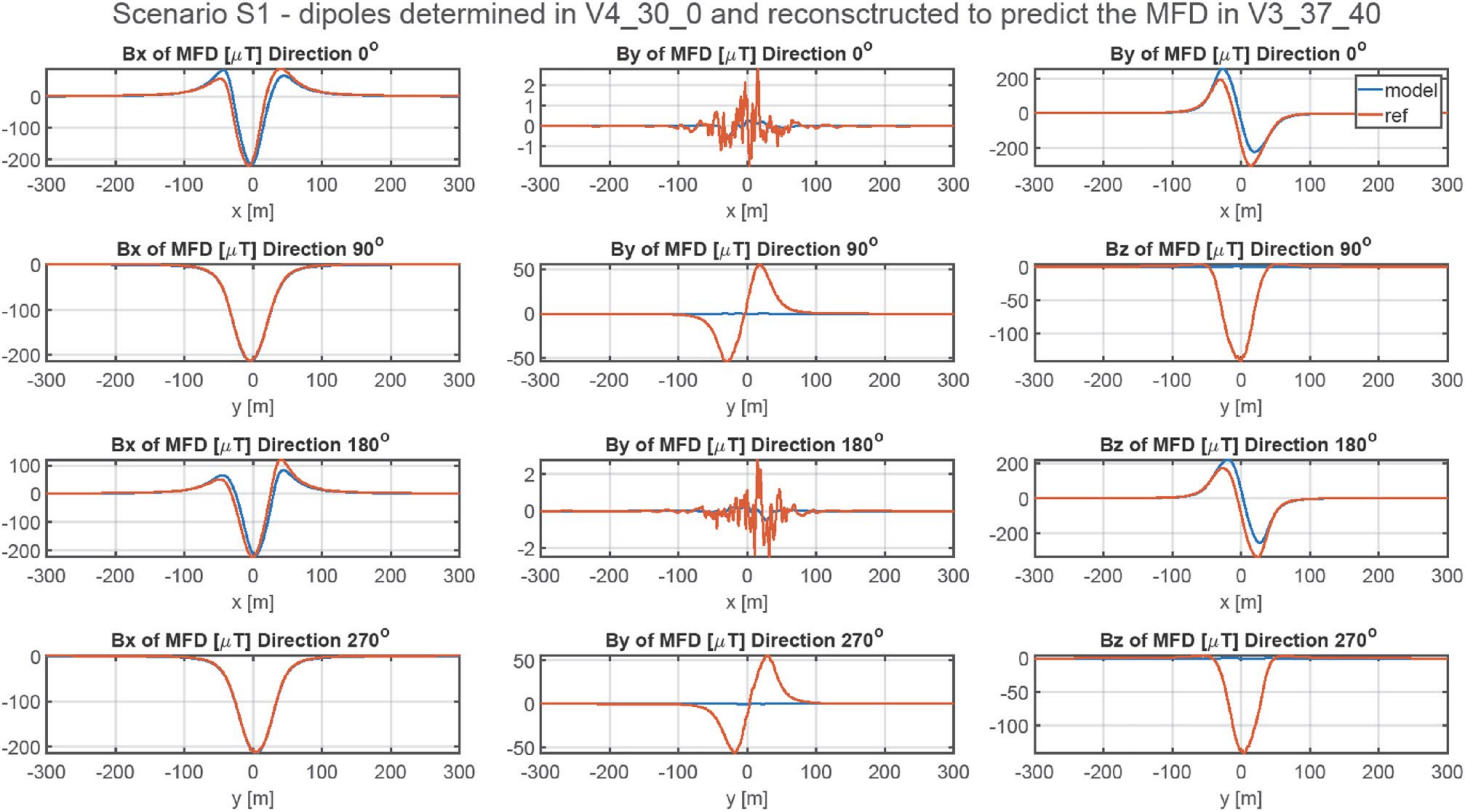
Validation of magnetic signatures at point V3 based on scaled multi-dipole parameters obtained for point V4.

The ship’s magnetic signatures without permanent magnetization were calculated in Opera. The parameters of the multi-dipole model for each point V1 ÷ V6 were obtained from the data for four ship courses. After scaling the induced moments of the multi-dipole model, the magnetic signatures were compared at all points with those obtained from Opera. The magnetic signatures calculated in four directions under the ship keel (*z* = − 20 m) are available, for all cases, at^27^. Figure 8 presents one of these signatures: REF_1_VER_3. For this case, the RMS errors are less than 0.9 nT, and the maximum absolute errors for four directions are less than 4.8 nT. Another signature: REF_3_VER_5 is presented in Fig. 9. For this case, the RMS errors are less than 0.6 nT, and the maximum absolute errors for four directions are less than 4.72 nT. The reconstructed magnetic signatures for the two presented cases are very correct. The RMSE and MaxAbsError indicators obtained for all investigated cases are given in Tables 4 and 5, respectively. The errors of the reconstructed magnetic signatures which were obtained after scaling the multi-dipole model at three special points (V2, V4, V6) are unacceptable. The magnetic signatures at point V3 which were reconstructed based on the scaled multi-dipole model achieved at point V4 (Equator) are shown in Fig. 10. At point V2 and V6 (geographic poles), the horizontal component of the Earth magnetic vector field is null, while at point V4, only the horizontal component of the magnetic field is present. For this reason, correct determination of model parameters with the presented method is not possible for these cases. The conclusion is that the magnetic data at these points does not allow correct calculation of dipole parameters and the reconstruction of ship signatures in other places.

**Table 4:** RMSE of Scenario 1.

	V1 VER	V2 VER	V3 VER	V4 VER	V5 VER	V6 VER
V1 REF	0.38863	0.33295	0.80769	0.43673	0.50444	0.42554
V2 REF	25.36511	0.32493	41.92127	44.49641	40.79002	0.41533
V3 REF	0.43610	0.34750	0.73514	0.53781	0.58818	0.44404
V4 REF	42.18998	47.14489	21.37385	0.42467	42.78226	60.16733
V5 REF	0.38850	0.33522	0.80796	0.43204	0.50223	0.42843
V6 REF	25.36493	0.31397	41.92119	44.49635	40.78988	0.40133

**Table 5:** Maximum absolute error of Scenario 1.

	V1 VER	V2 VER	V3 VER	V4 VER	V5 VER	V6 VER
V1 REF	4.47677	3.58545	4.75498	3.35791	4.78479	4.57573
V2 REF	154.92046	3.45414	257.44145	272.96322	251.24838	4.40818
V3 REF	4.46037	3.62191	4.69025	3.34646	4.71532	4.62226
V4 REF	275.36280	307.59994	140.00532	3.12019	280.16875	392.56511
V5 REF	4.45171	3.63922	4.65805	3.30087	4.65898	4.64434
V6 REF	155.05351	3.34625	257.50612	272.95842	251.06978	4.27371

Note that the cells in the table filled in green mean that the corresponding combination of reference data vs. verification data is considered a correct reproduction, while the unfilled (white) cells represent the scenarios considered unsuccessful.

### Methodology of validation of ship’s magnetic reconstruction with permanent magnetization

**Figure 11:**
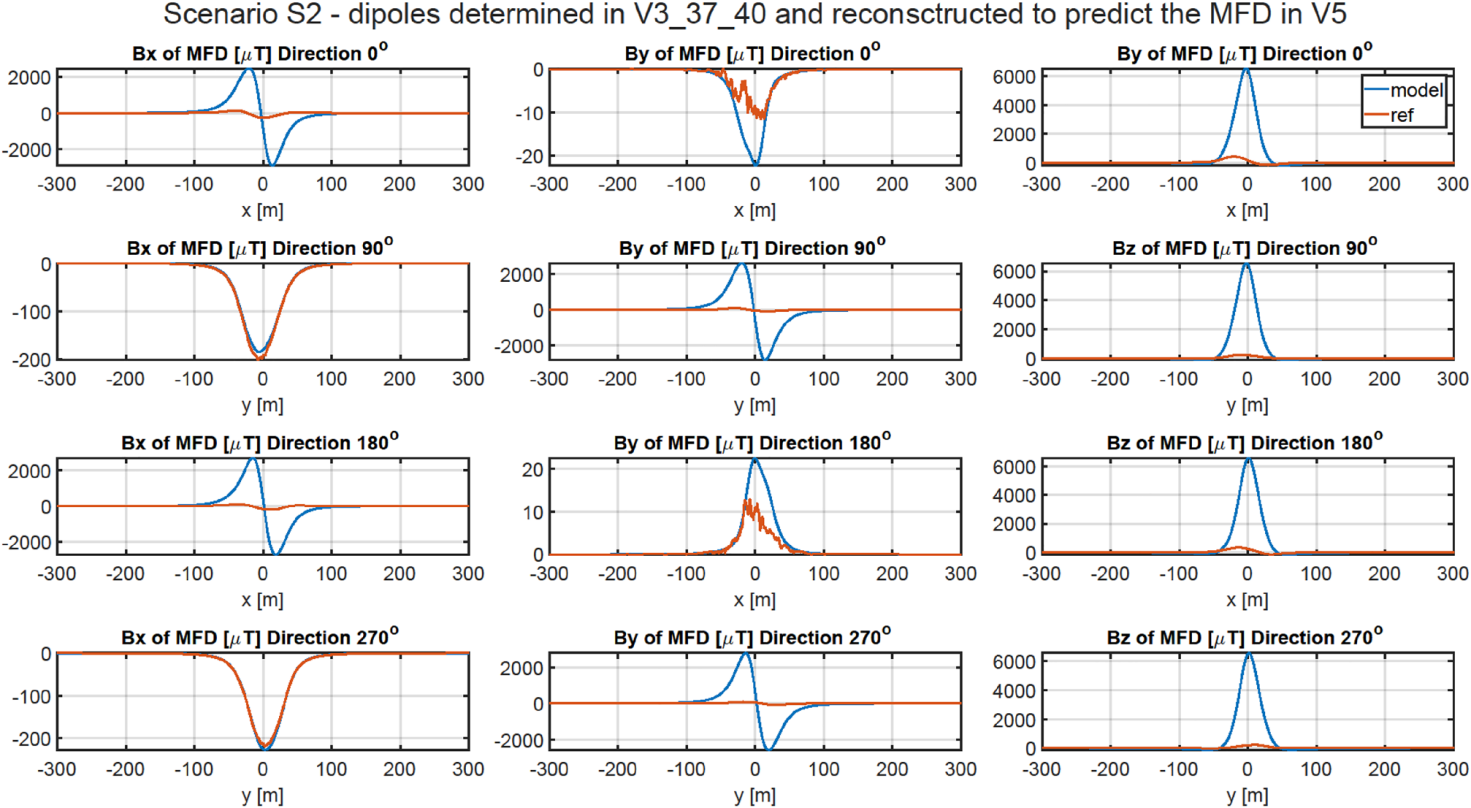
Validation of magnetic signatures at point V5 based on scaled multi-dipole parameters obtained for point V3.

A more complicated, but also more practical problem concerns permanent magnetization of the ship. The magnetic signatures for four directions and for all points V1 ÷ V6 are available at^27^. The RMSE and MaxAbsError indicators for all cases are given in Tables 6 and 7, respectively. The magnetic signatures reconstructed based on the scaled multi-dipole models are unacceptable at all points. The reconstruction of the magnetic signatures at point V3 after scaling the multi-dipole model obtained at point V5 is shown in Fig. 11. Qualitative and quantitative differences in the magnetic signatures are enormous. The performed analysis of the reconstruction of ship’s magnetic signatures without vertical permanent magnetization has shown that the vertical components of the induced and permanent dipoles are mixed. It is therefore necessary to provide the multi-dipole model with the magnetic data for two different vertical components of the external field.

**Table 6:** RMSE of Scenario 2.

	V1 VER	V2 VER	V3 VER	V4 VER	V5 VER	V6 VER
V1 REF	0.40718	34.00250	143.06628	289.70422	583.38340	702.81155
V2 REF	38.40973	0.34506	154.92390	276.01434	520.94357	619.73785
V3 REF	279.17762	345.54031	0.77643	286.18402	859.31763	1092.38201
V4 REF	42.18395	47.13882	21.36811	0.45687	42.78859	60.17350
V5 REF	793.48209	839.73907	598.90738	399.44798	0.51986	162.44964
V6 REF	702.68753	736.19203	560.91233	415.24371	126.32590	0.42718

**Table 7:** Maximum absolute error of Scenario 2.

	V1 VER	V2 VER	V3 VER	V4 VER	V5 VER	V6 VER
V1 REF	4.09196	257.42416	1085.59094	2196.06836	4419.57591	5321.64266
V2 REF	268.18391	3.36620	1172.39268	2100.95255	3956.96591	4688.99555
V3 REF	2062.42706	2551.55382	4.59067	2114.96135	6347.07297	8066.63016
V4 REF	275.82139	307.56707	141.19544	3.75895	280.79650	392.66487
V5 REF	5640.98650	5968.38447	4259.11696	2841.79833	5.59815	1153.62255
V6 REF	5410.55444	5654.41983	4323.75620	3204.85324	961.86852	4.81944

### Methodology of validation of ship’s magnetic reconstruction with permanent magnetization using two vertical components of the external field

**Figure 12:**
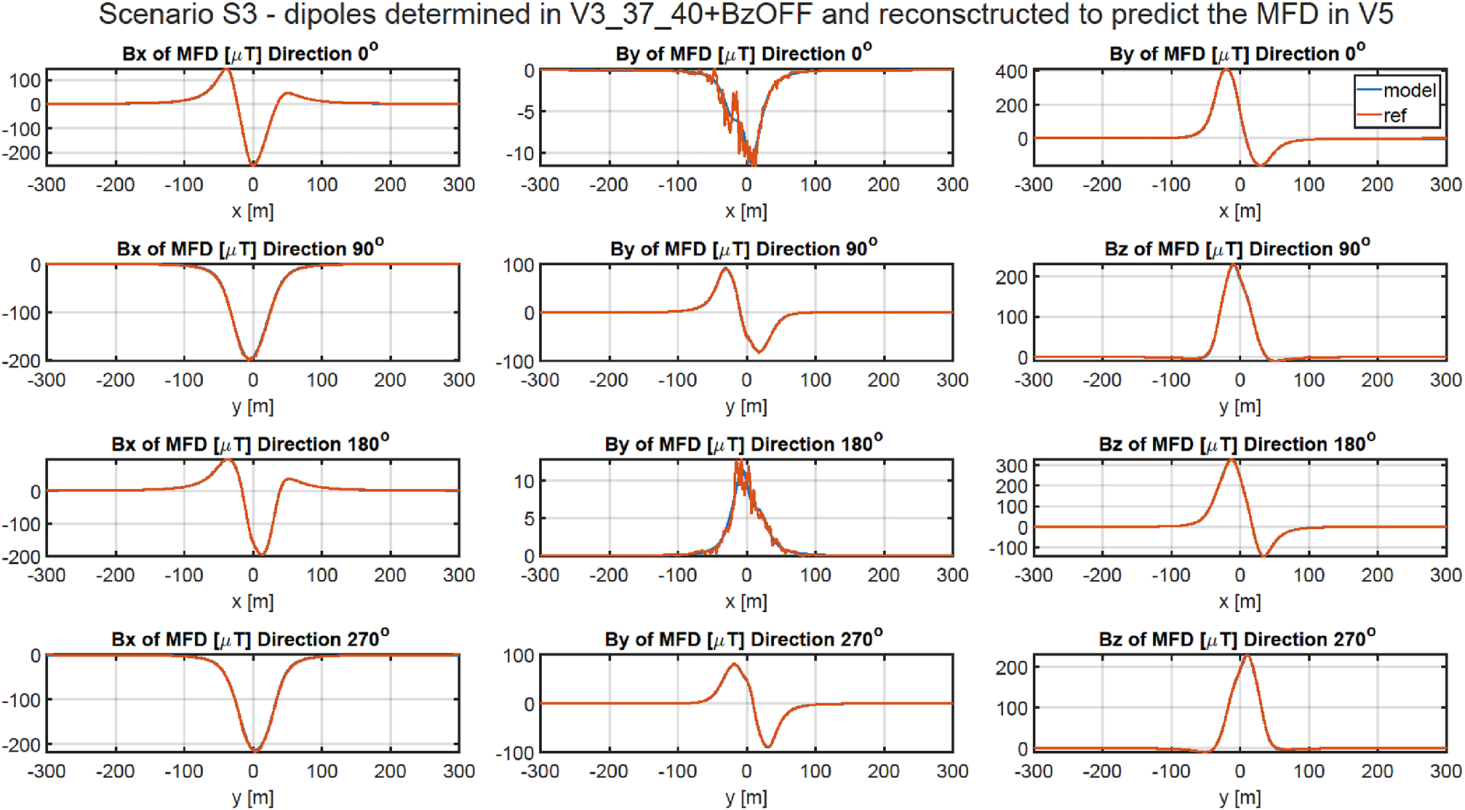
Validation of magnetic signatures at point V5 based on scaled multi-dipole parameters obtained for point V3.

**Figure 13:**
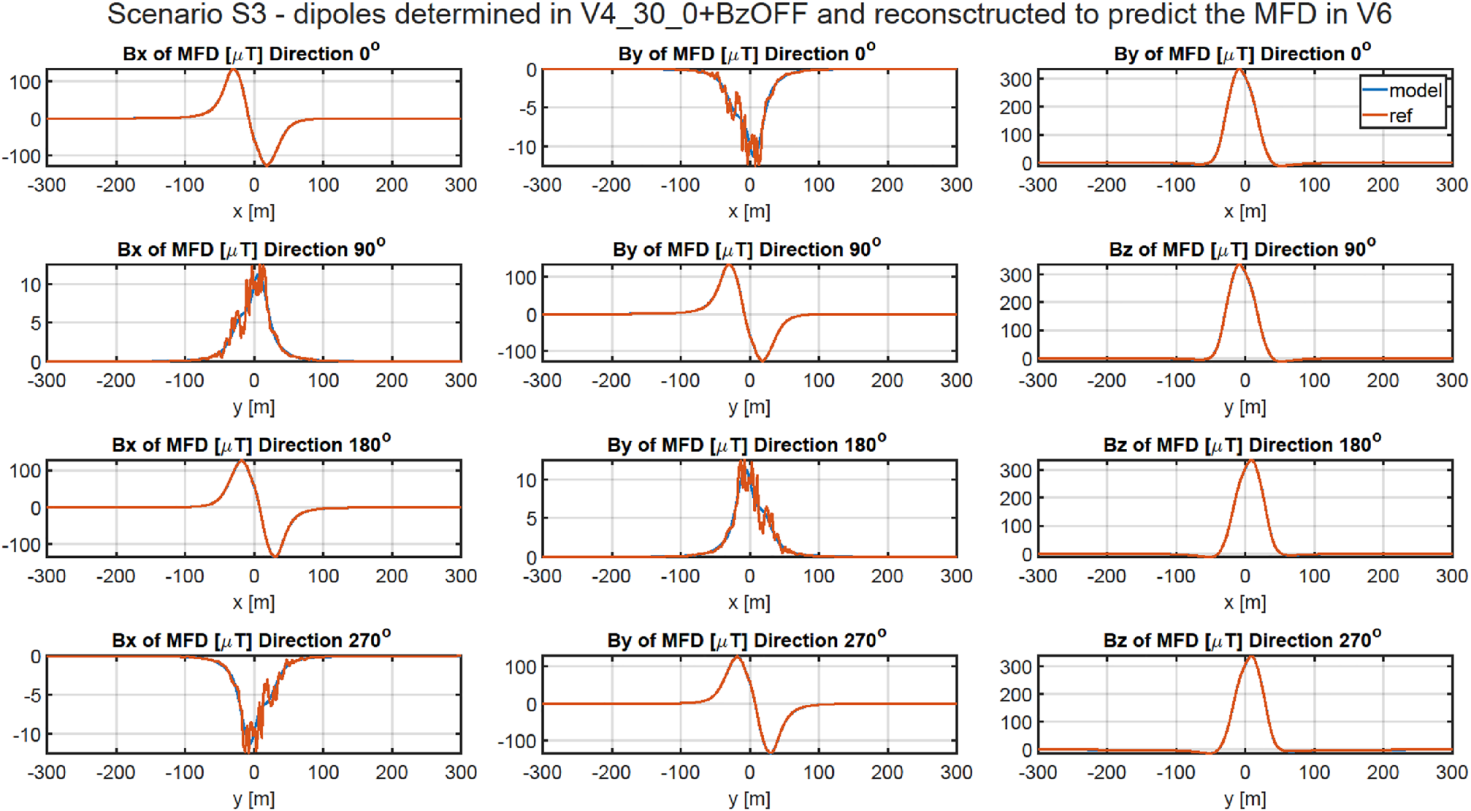
Validation of magnetic signatures at point V6 based on scaled multi-dipole parameters obtained for point V4.

As shown in Chapter 4.2, the vertical components of the permanent magnetization dipoles cannot be correctly designated when the magnetic data is acquired with only one value of the constant vertical component of the external field. A correct approach in this case is to acquire the magnetic data in four magnetic directions for two different values of this component. Therefore, in addition to the data about the vertical component of the Earth’s magnetic field already collected at a given geographical location in four magnetic directions, additional data must be acquired for a different value of this component. Change in the value of the vertical component of the Earth's magnetic field can be executed by using a system of constant current coils generating a uniform vertical magnetic field around the vessel. In all cases presented in this Chapter, the magnetic data was added for the vertical component of the Earth field intensity changed by 20 A/m. The magnetic signatures for all cases are available at^27^. The RMS and MaxAbsError indicators for all cases are given in Tables 8 and 9, respectively. The multi-dipole model set out at points V1, V3, V4, and V5 allows to reconstruct the ship’s magnetic signatures with a high degree of accuracy. The magnetic signatures at point V3 reconstructed based on the scaled model obtained at point V5 are shown in Fig. 12. For this case, the RMS errors are less than 0.66 nT, and the maximum absolute errors for four directions are less than 5.72 nT. The magnetic signatures at point V6 reconstructed based on the scaled model obtained at point V4 are shown in Fig. 13. For this case, the RMS errors are less than 0.45 nT, and the maximum absolute errors for four directions are less than 4.8 nT. The reconstructed magnetic signatures for the two presented cases are very correct.

**Table 8:** RMSE of Scenario 3.

	V1 VER	V2 VER	V3 VER	V4 VER	V5 VER	V6 VER
V1 REF	0.39888	0.34522	0.81383	0.51858	0.58416	0.55094
V2 REF	25.36521	0.33900	41.92138	44.49717	40.79093	0.53255
V3 REF	0.49436	0.44184	0.78555	0.59623	0.66386	0.61041
V4 REF	0.40522	0.35480	0.81631	0.47551	0.51798	0.44896
V5 REF	0.49142	0.46076	0.84247	0.47725	0.51766	0.45448
V6 REF	25.36954	0.59121	41.92291	44.49698	40.79022	0.44601

**Table 9:** Maximum absolute error of Scenario 3.

	V1 VER	V2 VER	V3 VER	V4 VER	V5 VER	V6 VER
V1 REF	4.21768	3.33234	4.45003	5.01792	5.74783	5.08253
V2 REF	156.56497	3.23927	258.46447	273.34370	251.01810	4.46255
V3 REF	4.35148	3.42078	4.63594	5.21144	5.71749	4.93766
V4 REF	4.16928	3.31085	4.43749	4.60111	5.42800	4.77724
V5 REF	4.65822	3.77411	4.82967	4.55913	5.63883	5.00773
V6 REF	159.89117	4.60323	261.02393	273.52957	250.81823	4.79205

**Figure 14:**
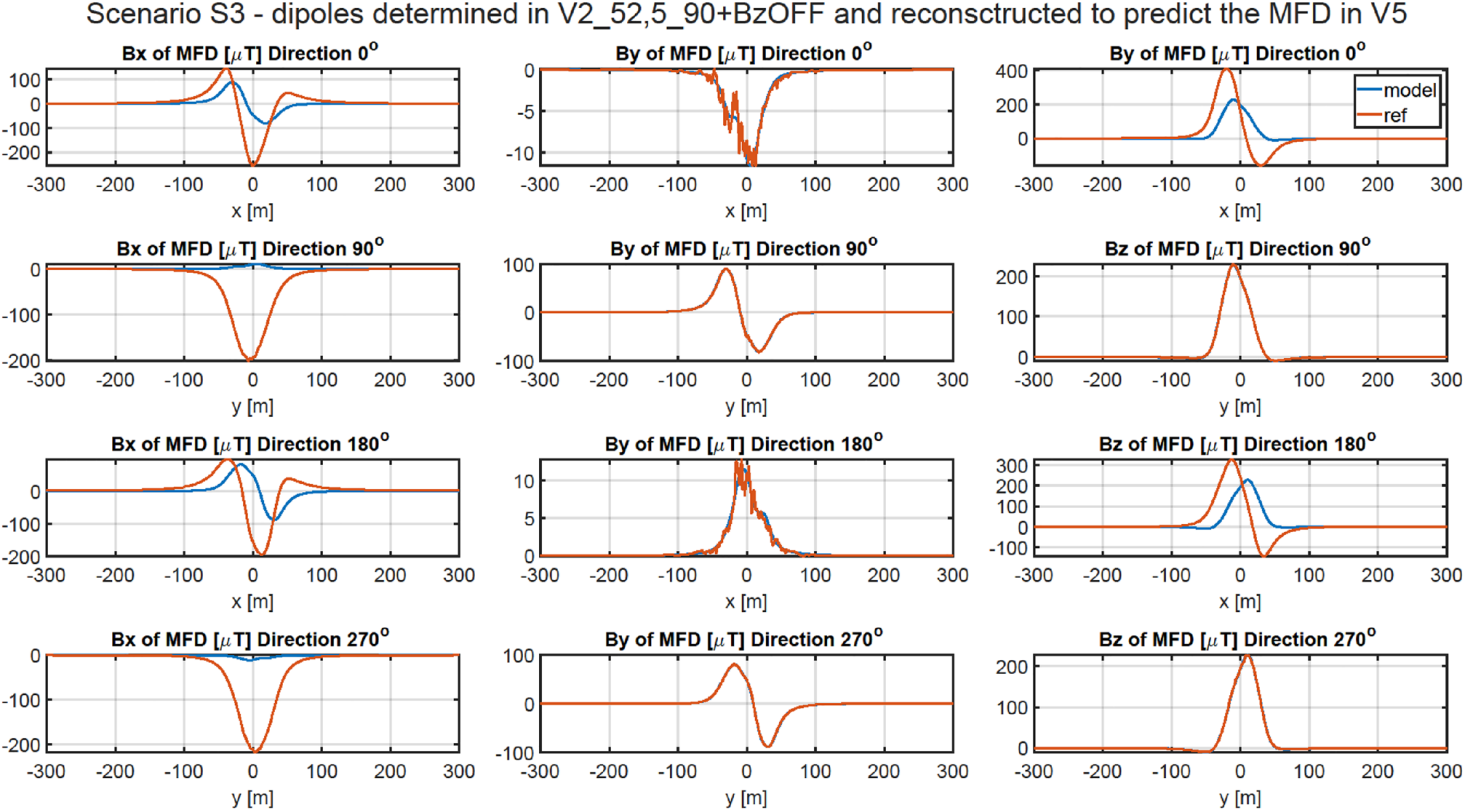
Validation of magnetic signatures at point V5 based on scaled multi-dipole parameters obtained for point V2.

The magnetic signatures at point V5 reconstructed based on the scaled model obtained at point V2 are shown in Fig. 14. For this case, the RMS errors are above 40 nT, and the maximum absolute errors for four directions are above 251 nT. As it can be seen in Tables 8 and 9, the signatures determined based on the data at points V2 and V6 (geographic poles) are not correct.

## Methodology of validation of ship’s magnetic reconstruction with permanent magnetization for an arbitrarily selected depth and/or direction using two vertical components of external field

The multi-dipole model has the ability to reconstruct magnetic signatures for an arbitrary measurement direction and depth, not only for the directions and depths that were used when teaching the model, i.e., determining dipole parameters. Combining the ability, described in the article, to reconstruct the signature in a different geographic location than that in which the dipole parameters were determined with the ability to use an arbitrarily chosen depth and direction, a set of universal functions of the multi-dipole model is obtained. To illustrate the described properties, additional data sets were generated by the FEM with already known positions, but for other depths and directions of ship’s position. Details are shown in Table 10.

**Table 10:** Geographic position and values of magnetic field density at validation points.

Point	Geographic position Latitude [°], Longitude [°]	Total ambient field module [µT]	Inclination [°]	*B*_*Ex*_ [μT]	*B*_*Ey*_ [μT]	*B*_*Ez*_ [μT]	Direction [°]	Depth [m]
V1	55 N, 2 E	50	70°	17.101	0.000	− 46.985	210	− 35
V2	90 N, 165 E	52.5	90°	0.000	0.000	− 52.500	0	− 27
V3	15 N, 100 W	37	40°	28.343	0.000	− 23.783	45	− 25
V4	10 N, 30 W	30	0°	30.000	0.000	0.000	120	− 31
V5	25 S, 100 E	55	− 60°	27.500	0.000	47.631	300	− 30
V6	63 S, 135 E	67	− 90°	0.000	0.000	67.000	0	− 23

**Figure 15:**
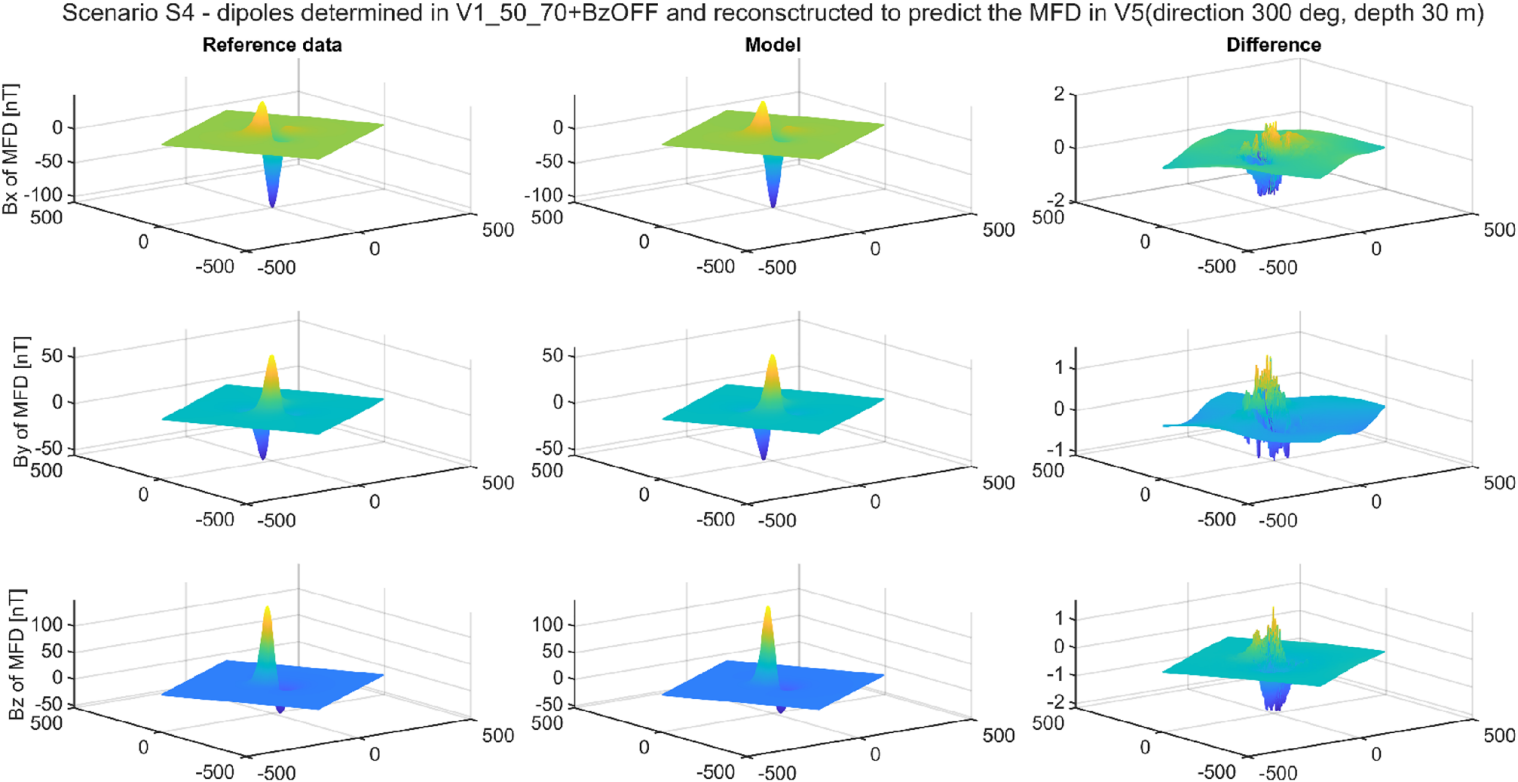
Validation of magnetic signatures at point V5 based on scaled multi-dipole parameters obtained for point V1.

**Figure 16:**
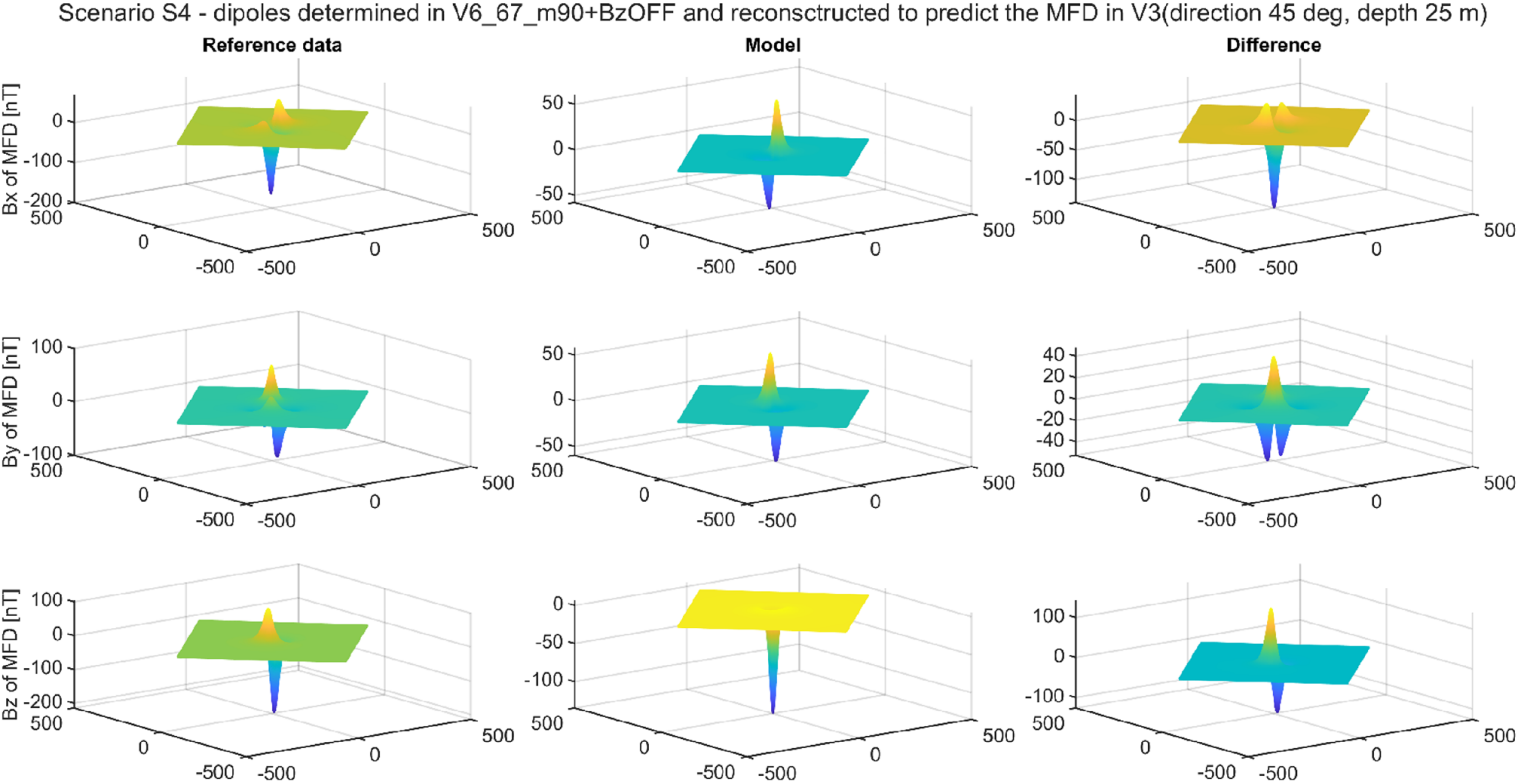
Validation of magnetic signatures at point V3 based on scaled multi-dipole parameters obtained for point V6.

In this simulation calculation, the values of the dipole parameters determined for Scenario 3 (BZ_off) were used. In addition to these dipoles, the direction and depth parameters from Table 9 were introduced into the model. The results were calculated for the entire fields, and not only for the paths. This form of verification and data presentation provides even more certainty, as the entire area of magnetic anomaly can be evaluated. The RMSE results are given in Table 10, and the maximum absolute error values in Table 11. Figure 15 shows the reconstruction which has been successfully completed, while Fig. 16 shows the reconstruction that failed. Analysing the data collected in Tables 11 and 12, it can be concluded that they coincide with the results obtained in Scenario 3. When the data for determining the model parameters come from points V1, V3, V4, and V5, then it is possible to map the signature for any other location, direction, and/or depth. Due to the impossibility of determining the magnetic field components Bx and By, the data from the Poles, i.e., points V2 and V6 allows the reproduction of signatures only at these Poles.

**Table 11:** RMSE of Scenario 4.

	V1DIR210DPTH35	V2DIR0DPTH27	V3DIR45DPTH25	V4DIR0DPTH31	V5DIR300DPTH30	V6DIR0DPTH23
V1 REF	0.11092	0.13288	0.34845	0.13422	0.25031	0.18953
V2 REF	3.13540	0.12823	7.84276	5.28386	5.09526	0.20852
V3 REF	0.14213	0.14905	0.37365	0.21327	0.27947	0.14391
V4 REF	0.10841	0.12341	0.33769	0.11671	0.22518	0.12973
V5 REF	0.11262	0.13033	0.33468	0.11204	0.21535	0.10418
V6 REF	3.17229	0.16922	7.82050	5.28934	5.09940	0.10659

**Table 12:** Maximum absolute error of Scenario 4.

	V1DIR210DPTH35	V2DIR0DPTH27	V3DIR45DPTH25	V4DIR0DPTH31	V5DIR300DPTH30	V6DIR0DPTH23
V1 REF	1.04390	2.63409	2.69585	1.58831	2.14450	3.33181
V2 REF	44.86862	2.58162	141.02476	86.62098	85.82288	4.19840
V3 REF	1.67361	2.42181	3.08910	2.17422	2.57356	3.90420
V4 REF	1.10667	2.64425	2.69267	1.32814	1.86488	3.32207
V5 REF	1.23987	2.65738	2.86639	1.37470	1.71647	3.44440
V6 REF	44.98469	2.44705	141.08415	86.60542	85.91737	3.24342

## Conclusions

A new universal approach to the reconstruction of ship’s magnetic signatures is described in the article. In this approach, the magnetic signatures of the ship are first acquired on the measuring range in four magnetic directions. After subtraction from the Earth magnetic field, the measured three components of the total magnetic field give the own magnetic field of the ship. Based on the measurements taken, the ship’s magnetic field can be replaced by the magnetic field of the virtual multi-dipole model, which allows to reconstruct the ship magnetic field for any course and for depths larger than the measuring depth. With the use of two different values of the vertical component of the external magnetic field, the values of the ship’s magnetic field vector components measured in four magnetic directions allow the parameters of the induced and permanent dipoles to be correctly determined. Once the induced dipoles of the multi-dipole model are appropriately rescaled, it is possible to accurately reconstruct the magnetic signatures of a ship situated anywhere on Earth. This method requires an arrangement of special coils with constant current to change the vertical component of the earth's magnetic field.

The measurements of the ship’s magnetic field should not be made at the magnetic poles, because in these places it is difficult to reproduce the parameters of the multi-dipole model without a special coil system changing the horizontal components of the Earth magnetic field. Verification studies were conducted with synthetic data based on locations at various points on Earth. With the exception of points with features completely devoid of a horizontal component (magnetic poles), at all other points it was possible to successfully determine the dipole parameters and then use them to reconstruct the signature at any geographic position, ship course, and depth lower than the measuring depth. Thus, the versatility of the multi-dipole model has been confirmed through numerical experiments. It would be very fruitful to confront the obtained results with real field measurements.

An archive with a set of input and verification data is shared by the authors for further analysis^27^. Any researcher can use the source data to verify the research results presented in the article and to search for new optimization algorithms in determining the parameters of the multi-dipole model. The dataset contains source synthetic magnetic data for a numerical model of a corvette-type ship. The data are provided for 6 locations around the world with different values of the Earth's magnetic field V1 ÷ V6. The attached data are in Matlab format. MAT, but the data can also be used in Octave software.

## Data Availability

The datasets generated during and/or analysed during the current study are available in the Magnetic signature reproduction of ferromagnetic ships at arbitrary geographical position, direction and depth using a multi-dipole model—source and verification dataset with description (synthetic magnetic data.zip) repository, https://mostwiedzy.pl/en/open-research-data/magnetic-signature-reproduction-of-ferromagnetic-ships-at-arbitrary-geographical-position-direction-,102011752152696-0.
